# P-1640. Antibiotic prescribing guideline-concordance for sinusitis in adult and pediatric primary care practices

**DOI:** 10.1093/ofid/ofae631.1806

**Published:** 2025-01-29

**Authors:** Lauren Dutcher, Graciela Gonzalez-Hernandez, Sara Rendell, Lacey M Serletti, Mickael Boustany, Karen O’Connor, Davy Weissenbacher, Leigh Cressman, Jeffrey S Gerber, Robert Grundmeier, Keith W Hamilton

**Affiliations:** University of Pennsylvania Perelman School of Medicine, Philadelphia, Pennsylvania; Cedars-Sinai Medical Center, West Hollywood, California; Hospital of the University of Pennsylvania, Philadelphia, Pennsylvania; University of Pennsylvania Perelman School of Medicine, Philadelphia, Pennsylvania; Children's Hospital of Philadelphia, Philadelphia, Pennsylvania; University of Pennsylvania, Philadelphia, Pennsylvania; Cedars-Sinai Medical Center, West Hollywood, California; University of Pennsylvania Perelman School of Medicine, Philadelphia, Pennsylvania; Children's Hospital of Philadelphia, Philadelphia, Pennsylvania; Children's Hospital of Philadelphia, Philadelphia, Pennsylvania; University of Pennsylvania Perelman School of Medicine, Philadelphia, Pennsylvania

## Abstract

**Background:**

Antibiotics are only sometimes indicated for acute sinusitis, a commonly diagnosed infection in the ambulatory setting and an important target for antibiotic stewardship. However, assessment of the appropriateness of antibiotic use for sinusitis is based on clinical signs and symptoms, and therefore requires manual chart abstraction. We present a study assessing the appropriateness of antibiotic prescribing in adult and pediatric sinusitis visits.

**Figure:**
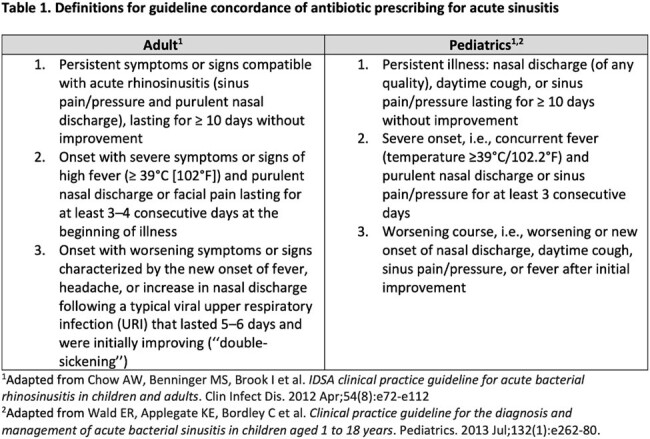

**Methods:**

Outpatient encounters for sinusitis in adult and pediatric primary care practices within two health systems with an antibiotic prescribed from July 1, 2017 through June 30, 2021 were identified by ICD-10 code (J01 and J32). Of these, 600 encounters were randomly selected for review (300 from adult practices; 300 from pediatric practices). Five trained physician annotators reviewed prescriber notes from these encounters and identified relevant signs/symptoms, duration of illness, and disease trend and severity; annotators used these findings to assess overall guideline concordance of antibiotic prescribing using definitions adapted from clinical guidelines (table 1). Two physicians performed adjudication for disagreements (LD, KWH).

**Figure:**
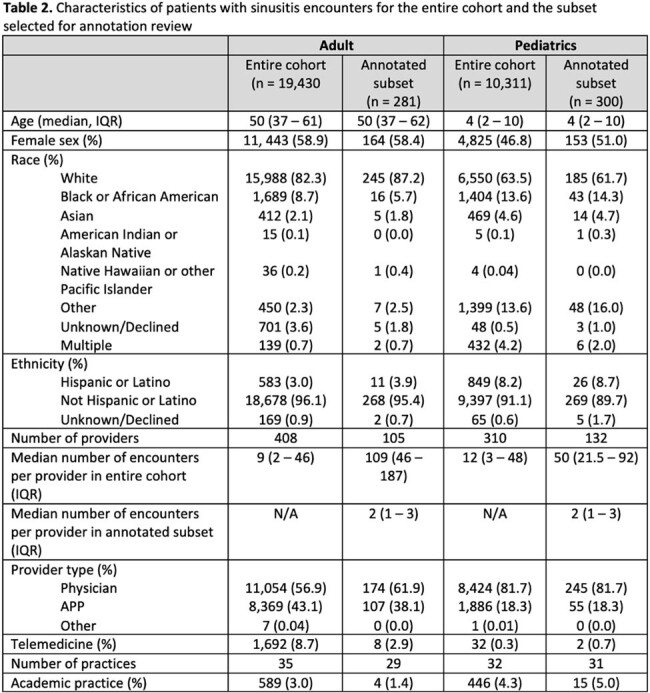

**Results:**

Demographics and characteristics of included encounters are reported in Table 2. In adults, 19 encounters were excluded after review due to a note being unrelated to the sinusitis encounter; of the remaining 281, 37 (13.2%) were considered guideline concordant, 157 (55.9%) were considered not guideline-concordant, and 87 (31.0%) were potentially guideline concordant but with insufficient information. In pediatrics, 184 (61.3%) were considered guideline concordant, 99 (33.0%) were considered not guideline-concordant, and 17 (5.7%) were potentially guideline concordant but with insufficient information. Common reasons for guideline non-concordance are described in Table 3.

**Figure:**
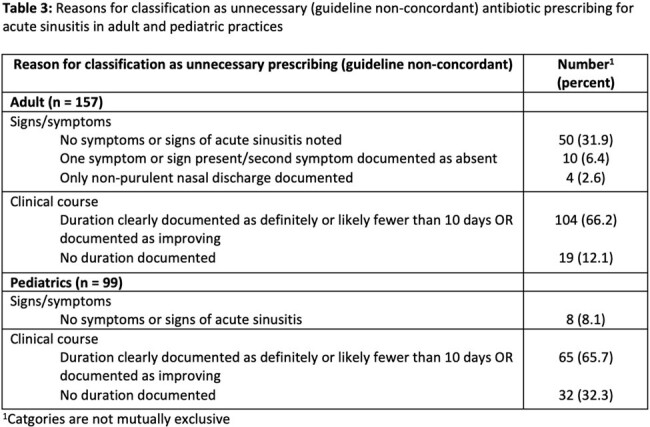

**Conclusion:**

Antibiotic prescribing forsinusitis was frequently not concordant with guidelines. Prescribing was more frequently concordant in pediatric compared to adult-care settings. In both groups, duration of symptoms was frequently documented as fewer than 10 days, suggesting a potential target for antibiotic stewardship interventions.

**Disclosures:**

**All Authors**: No reported disclosures

